# EXAMINING AGE, RACE, AND COMMUNITY ON PREVENTIVE HEALTH AND COVID-19 VACCINATION AMONG ADULTS 65+

**DOI:** 10.1093/geroni/igac059.796

**Published:** 2022-12-20

**Authors:** Taylor Patskanick, Sophia Ashebir, Alexa Balmuth, Sophia Lee, Joie Le, Lisa D'Ambrosio, Joseph Coughlin

**Affiliations:** Massachusetts Institute of Technology, Somerville, Massachusetts, United States; Massachusetts Institute of Technology, Cambridge, Massachusetts, United States; Massachusetts Institute of Technology, Cambridge, Massachusetts, United States; Massachusetts Institute of Technology, Cambridge, Massachusetts, United States; Massachusetts Institute of Technology, Cambridge, Massachusetts, United States; Massachusetts Institute of Technology, Cambridge, Massachusetts, United States; Massachusetts Institute of Technology, Cambridge, Massachusetts, United States

## Abstract

During the ongoing spread of COVID-19 and its variants, older adults remain an age group particularly at-risk for poorer health outcomes, not only related to infection with COVID-19, but also due to disruptions in access to preventive health services, including routine vaccination. In the U.S., older adults have generally had high uptake of the COVID-19 vaccines, but differences persist regionally and between older adults from minority racial backgrounds. The purpose of the following study was to better understand how groups of Black and white-identifying adults ages 65+ described the impact of the COVID-19 pandemic on their preventive health behavior and healthcare use, including what contributed to their decision to receive or not receive a primary COVID-19 vaccination series. Seventy-five participants were purposively sampled and stratified into virtual focus groups based on their age, racial identity, vaccination status, and relationship to a local community. Findings leverage data from a pre-group questionnaire and focus groups conducted in November 2021. Analyses revealed differences among sub-groups about how the pandemic has impacted their relationship to their local community. Participants described the extent of the pandemic’s disruption to their healthcare access, including modifications to in-person care, use of telehealth, and engagement in new health behaviors. Decision-making related to the COVID-19 vaccine differed among the vaccinated and unvaccinated and white and Black-identifying groups, including factors related to interpersonal and systemic trust, independent research, and bodily autonomy. Implications of this research for public health and practitioners working with older adults will be discussed.

